# Natural variation of respiration-related traits in plants

**DOI:** 10.1093/plphys/kiac593

**Published:** 2022-12-22

**Authors:** Mustafa Bulut, Saleh Alseekh, Alisdair R Fernie

**Affiliations:** Department of Root Biology and Symbiosis, Max-Planck-Institute of Molecular Plant Physiology, Am Mühlenberg 1, Potsdam-Golm 14476, Germany; Department of Root Biology and Symbiosis, Max-Planck-Institute of Molecular Plant Physiology, Am Mühlenberg 1, Potsdam-Golm 14476, Germany; Center for Plant Systems Biology and Biotechnology, Plovdiv 4000, Bulgaria; Department of Root Biology and Symbiosis, Max-Planck-Institute of Molecular Plant Physiology, Am Mühlenberg 1, Potsdam-Golm 14476, Germany; Center for Plant Systems Biology and Biotechnology, Plovdiv 4000, Bulgaria

## Abstract

Plant respiration is one of the greatest global metabolic fluxes, but rates of respiration vary massively both within different cell types as well as between different individuals and different species. Whilst this is well known, few studies have detailed population-level variation of respiration until recently. The last 20 years have seen a renaissance in studies of natural variance. In this review, we describe how experimental breeding populations and collections of large populations of accessions can be used to determine the genetic architecture of plant traits. We further detail how these approaches have been used to study the rate of respiration per se as well as traits that are intimately associated with respiration. The review highlights specific breakthroughs in these areas but also concludes that the approach should be more widely adopted in the study of respiration per se as opposed to the more frequently studied respiration-related traits.

## Teaser

While most respiration-related studies accessing natural variation focus on respiratory metabolites, novel high-throughput techniques to capture respiration rates will enable novel discoveries.

## Variation in respiration rates of plants

Plant respiration represents one of the greatest global metabolic fluxes with respiration rates typically being 40%–60% of the rate of carbon assimilation (the highest global metabolic flux; (Amthor, 1994; Gifford, 1994)). As such, global plant respiration represents about half of the annual input of CO_2_ to the atmosphere from terrestrial ecosystems (Tilman et al., 2001). The exact rates, however, vary greatly between species, tissues, and developmental stages. Indeed, inter-specific rates of respiration have been found to vary by five orders of magnitude based on plant size and nitrogen content (Reich et al., 2006). Similarly, in illuminated leaves rates of respiration are estimated to be around 0.4 μmol m^−2^ s^−1^ whilst mitochondrially dense heterotrophic tissues such as potato tubers or intermediate tissues such as guard cells display respiration rates of considerably higher magnitude (Mawson, 1993; Atkin et al., 2000; Noguchi et al., 2001; Malone et al., 2006; Riewe et al., 2008). Moreover, the rate of plant respiration is massively influenced by temperature with mass standardized leaf respiratory rates varying several-fold among plant species at a given site and are, for example, three-fold higher, on average, in the Arctic than in the tropics (Atkin et al., 2015; O'Leary et al., 2019). The conquest of terrestrial ecosystems by plants was mainly achieved by adaptation in both molecular and metabolic optimization of energy balance/homeostasis (Kragh et al., 2017). As such, allocation and regulation of the major energetic product, such as sucrose, was a key in the evolution of flowering plants. During the emergence of spermatophytes, increased copy numbers of plastidic invertases correlated with rises in respiratory rates (Fernie et al., 2020). Whilst at an evolutionary level it is intriguing to note that rates of respiration in mosses are reported to be 0.000102–0.00196 μmol m^−2^ s^−1^, which is considerably lower than the rates in angiosperms, which are of the order of 0.72–0.77 μmol m^−2^ s^−1^ (Wright et al., 2004; Atkin et al., 2015). Finally, at a developmental level, it is well characterized that younger tissues have higher rates of respiration, for example at the shoot or root apical meristems, given that they need to sustain higher rates of growth (Ishihara et al., 2015; O’Leary et al., 2017). An extreme example of this is the ten-fold enhanced rate of respiration in the thermogenesis-mediated growth spurt of *Arum maculatum* spadices (Simon, 1959). However, other examples include oxygen and carbon dioxide partial pressures (Leakey et al., 2009) and damage due to herbivory (Holland et al., 1996). The above statements have largely been made on the classical view of respiration as an oxygen-consuming process on the basis of gas exchange measurements and therefore, must be caveated by the facts that the definite gas exchange composes of (1) non-respiratory carbon losses due to the synthesis of fatty acid and (2) consumption of O_2_ by non-respiratory oxidase activity (Sweetlove et al., 2013; O'Leary et al., 2019). It is important to note that where data is available regarding the biochemical pathways supporting respiration at either the transcript or even better, the metabolite level (O’Leary et al., 2020), these largely complement the statements made above. However, whilst certain explanations can be afforded to explain the large differences in respiration due to between species, developmental stages, or environmental effects. Studying at these levels often renders it difficult to go beyond descriptive studies and dissect the underlying mechanisms. For this purpose, the study of (1) intraspecific variation in respiration per se, (2) variance in the levels of respiratory intermediates, as well as (3) traits associated with respiratory changes are more appropriate. Whilst only a handful of approaches have comprehensively addressed the first of these (O'Leary et al., 2017; Scafaro et al., 2017; de Oliveira Silva et al., 2018), as described in the following two sections, many populations and collections of both model and crop species have been generated or collected that would be useful in this respect, since domestication inherently led to a reduction of allelic diversity among crop species (Fernie et al., 2006). The vast majority of modern crops are Angiosperms, which evolved and adapted their physiology and anatomy to allow higher capture of energy and carbon, elevating their species richness, and showcasing high natural variation when compared to wild relatives. Indeed, as we detail later in this article, some of them have already been used in the characterization of respiration or respiration associated traits.

## Natural variation as assessed from breeding populations

Targeted synthetic approaches, such as the introduction of foreign genes with higher efficacy and the recreation of ancestral genes (Scossa and Fernie, 2021), might be possible ways to improve organisms’ carbon household management. However, following a different route from the targeted approaches of transgenesis and marker-assisted mutagenesis, recent years have been characterized by a renaissance in the use of natural variance both in the pre-eminent model species Arabidopsis (*Arabidopsis thaliana*) (Kliebenstein et al., 2001; Koornneef et al., 2004; Weigel and Nordborg, 2005; Borevitz et al., 2007; Alonso-Blanco et al., 2009; Wu et al., 2018) and in an increasing number of crop species (Schauer et al., 2006; Alseekh et al., 2015; Wen et al., 2015; Chen et al., 2018; Di Vittori et al., 2021). Studies of metabolic traits in *Arabidopsis* have largely focused on understanding the principles underlying metabolic regulation and the influence of metabolism on growth and development (Kliebenstein et al., 2001; Keurentjes et al., 2006; Lisec et al., 2008, 2009; Rowe et al., 2008; Sulpice et al., 2009; 2010; Meyer et al., 2012) whilst those on crops have focused both on harvest index and yield (Schauer et al., 2006; Riedelsheimer et al., 2012a, 2012b) as well as on specific metabolites of particular importance in our various major crops (for example sugars and acid in tomato (*Solanum lycopersicum*), (Schauer et al., 2006; Rothan et al., 2019); starch, oil and 2,4-dihydroxy-7-methoxy-1,4-benzoxazin-3-one in maize (*Zea mays*), (Zhang et al., 2021) and flavones and terpenes in rice (*Oryza sativa*) (Peng et al., 2017; Zhan et al., 2020)). As for all quantitative traits, those associated with metabolism are characterized by continuous variation. In essence, the establishment of the genetic basis of quantitative traits commonly referred to as quantitative trait loci (QTL) has often been hampered due to their complex multigenic inheritance and strong interactions with the environment (Fernie and Klee, 2011). The principle of QTL mapping in segregating populations is based on parallel genotyping and phenotyping of progeny derived from a cross between distinct genotypes. Phenotypic values are then compared with molecular markers in the progeny in search of genomic regions displaying statistically significant associations of the trait of interest (Broman, 2001; Slate, 2005). Molecular marker technologies and next-generation sequencing have rendered this relatively facile even for the most complex traits.

**Figure 1:**
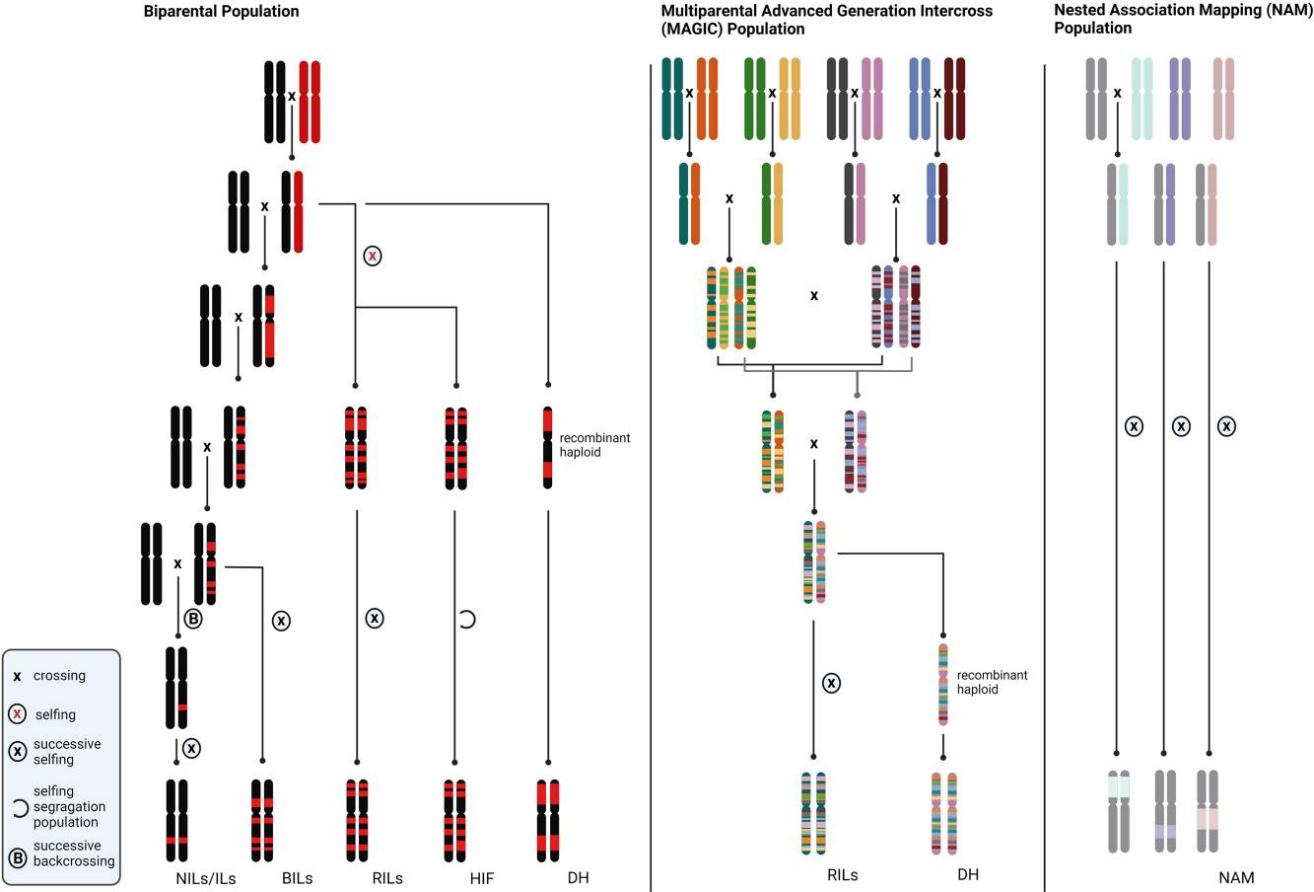
Schematic illustration of most commonly used QTL mapping populations. Laborious generated populations can mainly be distinguished by bi-parental (left) and multi-parental (mid, right) origin. Several different bi-parental populations can be generated, depending on the trait to assess and offspring self-compatibility, including near isogenic lines (NILs), introgression lines (ILs), backcrossed inbred lines (BILs), recombinant inbred lines (RILs) heterogeneous inbred family (HIF), and double haploids (DH). On the contrary, multi-parental lines, like multi-parental advanced generation inter-cross (MAGIC) populations are recombinant inbreds of multiple inter-crossed donors or NAM populations in which the donors are crossed to one “nested” genetic background.

QTL analysis makes use of the natural variation present within a species and has been successfully applied to various types of segregating populations (Figure 1). In plants, the use of “immortal” mapping populations consisting of homozygous individuals that can be propagated indefinitely is preferred since it permits replication and multiple analyses of the same population (Zamir, 2001). That said as we detail below heterogeneous inbred families (HIFs), also have utility in trait-gene analyses. As seen in the schematic representation in Figure 1 homozygous populations can be generated by repeated selfing, as is the case for recombinant inbred lines (RILs), but also by an induced chromosomal doubling of haploids, such as for doubled haploids (DHs) (Han et al., 1997; Rae et al., 1999; von Korff et al., 2004). RILs are likely advantageous over DHs since they are characterized by a higher frequency of recombination within the population, resulting from multiple meiotic events occurring during repeated selfing (Fernie and Klee, 2011). Another kind of immortal population is introgression lines (ILs) (Eshed and Zamir, 1994), which are obtained through repeated backcrossing and extensive genotyping. These are also sometimes referred to as near isogenic lines (NILs) (Monforte and Tanksley, 2000), or backcross inbred lines (BILs) (Jeuken and Lindhout, 2004; Blanco et al., 2006). However, BILs are actually more reminiscent of RILs in that they have a mosaic of donor and recurrent genomes rather than a single or at least a small number of chromosomal segment substitutions (see for example Ofner et al., (2016); Brog et al., (2019)). In plants, RILs and NILs are the most common types of experimental populations used for the analysis of quantitative traits. In both cases, the accuracy of QTL localization, referred to as mapping resolution, depends on population size. For RILs, the position of the recombination event is fixed and can therefore only be increased within the population by adding more lines, whilst in NIL populations resolution can be improved by minimizing the introgression size of each NIL (see for example Alseekh et al. (2013)). Consequently, to maintain genome-wide coverage either a larger number of lines or a high proportion of overlapping regions, or both, are needed.

Despite the similarities between these two types of mapping populations, large differences exist in their genetic makeup and the resulting mapping approaches utilized. Recombination frequency is in general higher in RIL populations than in equally sized NIL populations, allowing analysis of fewer individuals. Each RIL contains several introgressed fragments and, on average, each genomic region is represented by an equal number of both parental genotypes in the population (Fernie and Klee, 2011). Therefore, replication of individual lines is often not necessary because the effect of each genomic region on phenotypic traits is independently tested multiple times by comparing the two genotypic RIL classes. Moreover, the fact that RILs contain multiple introgressions per line can potentially reveal epistasis between loci. However, this may be deleterious with regard to the power to detect QTL. Furthermore, the wide variation of morphological and developmental traits among individuals within most RIL populations may hamper analysis of traits requiring uniformity of such traits (Fernie and Klee, 2011). By contrast, ILs preferably contain only a single introgressed segment per line, increasing the power to detect small-effect QTL. However, the presence of a single introgressed segment limits testing for genetic interactions and thereby the detection of epistatic QTL. Since most of the genetic background is identical for all lines, NILs show more limited developmental and growth variation, increasing the homogeneity across growth stages within experiments. BILs, largely possess the characteristics of RILs in that they display a mosaic of genomes thus combining both high resolution without replications (Szymański et al., 2020; Kazachkova et al., 2021) and the possibility to study epistasis (Fulop et al., 2016), with the advantages provided by the possibility of inter-specific crosses. The latter not being possible following the RIL breeding strategy due to the inability to self the F1 progeny due to sterility issues. As mentioned above, a final bi-parental derived population type that is commonly used is HIFs which are most frequently used in order to confirm QTL detected in a RIL population by taking a predecessor of a RIL that remains heterozygous for the region of interest but otherwise homozygous is selfed following a Mendelian manner segregation in the heterozygous region. This thus enables comparison of the trait of interest for that specific region for both parental genotypes in an isogenic background.

**Figure 2:**
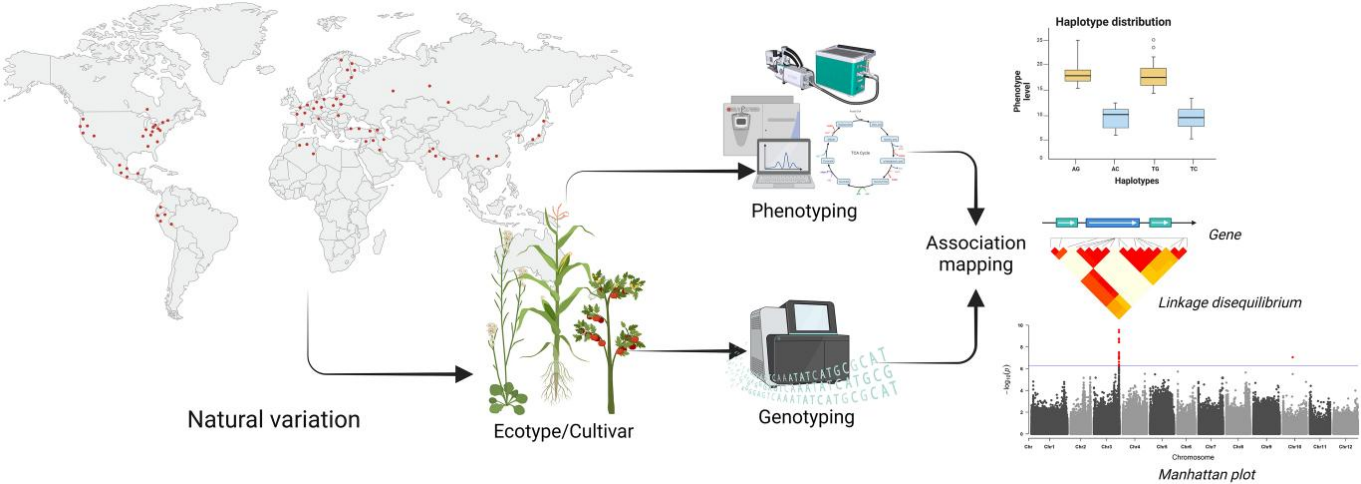
Schematic overview of accessing natural variation for GWAS. Fundamental for these kinds of studies is the presence of a population as diverse as possible to cover diversity in their genomic variants, like SNPs and structural variance (SV), as well as their phenotypic response, like metabolite levels and gas exchange rate. Both components are integrated to perform association mapping resulting in potential QTL detection. These QTL are constrained by determining the linkage disequilibrium for cosegregation and harbor in general candidate genes explaining the variance in the phenotypic response by comparing arithmetic mean over their haplotype distribution.

One disadvantage of the bi-parental approach is that it is limited by the allelic variance exhibited by the two parental lines. To circumvent this, the so-called multi-parental advanced generational inter-cross (MAGIC) populations (Cavanagh et al., 2008) were developed. There are many variants on this including nested association mapping (NAM) populations (Gage et al., 2020) and the recently developed Complete-diallel design plus unbalanced breeding-like inter-cross (CUBIC) populations (Liu et al., 2020). However, the basic principle of all remains the same, i.e. to maximize the allelic diversity present in the population in a manner, which allows high power genetic resolution. As a result, such populations often allow the mapping of trait variance to small genomic intervals and are not reliant on the allelic variance of a single pair of parents. Whilst the use of such populations to study respiration relevant traits is limited to date, given the strengths outlined above, their future adoption would seem to be a priority for research in this area.

## Genome-wide association mapping

Large differences in respiration render it challenging to study broad natural populations and in most cases, these do not exceed descriptive approaches in order to understand the underlying causes of metabolic regulations. An alternative, or complementary approach to that offered by breeding populations is that of directly assessing broad range natural variation of traits and associating this with differences in gene sequences via genome-wide association studies (GWAS) (Alseekh et al., 2021b). The aim of GWAS is highly simple—to detect the association between allele or genotype frequency and trait status. The first step is to select an appropriate study population considering both the size of the population and the amount of genetic and phenotypic variance that it possesses (Figure 2). Depending on how well the population has been studied, genotyping may be necessary and this is nowadays generally carried out either by single nucleotide polymorphism (SNP) arrays and imputation (Johnson et al., 2013) or by whole-genome sequencing (Tam et al., 2019). GWAS was pioneered by Klein et al. (2005), who identified a variant of the *Complement Factor H* gene as being strongly associated with age-related macular degeneration. Over the last 16 years, it has proven powerful in dissecting the genetic basis for variation in a range of complex phenotypes including disease in humans and animals and physiological and agronomic traits in plants (Huang et al., 2010; Tian et al., 2011, 2020; Horton et al., 2012; Chen et al., 2016; Tieman et al., 2017; Zhu et al., 2018). That said, it has been noted that population structure and unequal relatedness between individuals can result in spurious associations and thereby false discoveries. However, considerable effort has been made in order to combat this problem and to statistically account for population structure (Devlin and Roeder, 1999; Liu et al., 2016a, 2016b). An often cited criticism of GWAS concerns the fact that it often can only explain a modest proportion of heritability (Manolio et al., 2009), however, this would appear to be only a relatively small problem when the populations analyzed are large enough (Visscher et al., 2012; Ahlqvist et al., 2015). Although it is important to note that the population sizes currently used in plant science are generally far smaller than those defined as completely obviating such problems. Comparing the two genotypes mentioned above SNP arrays, offer the advantage that in addition to their lower cost, they are highly accurate with a well-established pipeline for analysis. By contrast, although less accurate and more expensive whole-genome sequencing provides coverage also of rare variants and even if the sample size is large enough for ultra-rare variants (Alseekh et al., 2021b). In addition, fine mapping is easier with whole-genome sequencing, however, these advantages come at the cost of higher computational costs including a higher multiple testing burden (Tam et al., 2019). To offset some of the limitations of SNP-based GWAS, sophisticated tools for genotype imputation have been developed which allow genotypes or untyped variants to be predicted. If the size of the reference panel is large enough and a subset is well sequenced this imputation has been demonstrated to be highly reliable (Marchini and Howie, 2010; Huang et al., 2015). However, whole-genome sequencing is the gold standard in GWAS (Steinthorsdottir et al., 2014; Fuchsberger et al., 2016; Lek et al., 2016) and has the potential to resolve many of the limitations of the method such as the identification of missed signals, accounting for population stratification, identification of ultra-rare mutations as well as gene–gene and gene–environment interactions and to explain even more of the missing heritability. As we described recently in an in-depth review on GWAS in plants, suitable collections now exist for a range of model, common, and even rare crop species as well as a range of non-cultivated species (Alseekh et al., 2021a, 2021b), rendering GWAS considerably more facile, than a mere few years ago. Given that breeding population and GWAS based approaches are highly complementary we will mix their discussion in the following trait-centric chapters.

## Natural variation in the rate of respiration per se

Given that the focus of this issue is plant respiration, the relative paucity of studies on respiration per se should not preclude them being discussed first before more indirect studies of respiration focused on respiratory metabolite levels or other diverse traits influenced by respiration or respiratory metabolites, respectively. That said, the situation is similar to that for photosynthesis in that very few reports cover the rate of respiration across broad natural variance. Fortunately, by contrast to the situation observed for photosynthesis (Sharwood et al., 2022; Theeuwen et al., 2022), the number of research papers in this field is still greatly in excess of the number of review articles. This review paper is specifically concerned with the natural variation of respiration. This is probably due to the fact that of the 11 studies summarized in Table 1 in which seven were published within the last five years. The earliest study we highlight that of Baxter et al. was published in 2005, however, it only focused on a subset of introgression lines—those previously characterized to display elevated Brix and only looked at transcript levels of respiratory enzymes (Baxter et al., 2005). In a similar vein but at the whole-genome level, Steinhauser et al. (2010) looked at the activities of several enzymes including enzymes of the tricarboxylic acid (TCA) cycle finding that many of these were determined by *trans*-QTL. More directly associated with respiration, Poorter et al. (2005) assessed gas exchange in a large F2 barley population finding several interesting QTL for photosynthesis but not for respiration with several similar studies being carried out in crossing populations of melon (Moreno et al., 2008; Ríos et al., 2017; Zarid et al., 2021). The first of these surprisingly revealed that NILs derived from two non-climacteric melon parental lines allowed the localization of a climacteric-ripening phenotype with enhanced rates of respiration (Moreno et al., 2008). The study of Rios et al. identified a NAC transcription factor to be responsible for the climacteric increase in respiration (Ríos et al., 2017), whilst transcriptomic analyses revealed 37 genes including many enzymes of the TCA cycle to be altered in a NIL with altered climacteric-ripening (Zarid et al., 2021). In 2018, de Oliveira Silva et al. (2018) assessed the genetic architecture of dark respiration in tomato leaves using the same tomato introgression lines as the studies on transcripts and enzyme activities described above allowing the identification of two QTL for dark respiration. Whilst dark respiration was also measured in rice GWAS and MAGIC populations (Qu et al., 2020; Xu et al., 2021) and root respiration was measured in a population of winter wheat (*Triticum aestivum*) (Guo et al., 2021). The rice GWAS study identified the *leucine-rich repeat receptor kinase 1* (LRK1) gene, as a major regulator of the rate of dark respiration (Qu et al., 2020), whilst the other study used dark respiration as a composite trait that correlates carbohydrate remobilization to nighttime temperatures (Xu et al., 2021). As stated above, the number of analyses is increased massively in recent years. Given the renaissance in regarding respiration as an important target for crop improvement (Amthor et al., 2019; Fernie et al., 2020), and the increasing number of miniaturized methods to measure respiration including the use of oxygen electrodes (Ast et al., 2012), microtiter plate reader respiration measurement protocols (Scafaro et al., 2017) and microscopic imaging-based approaches (Tschiersch et al., 2012), it is likely that in the next decade we will have access to an unprecedented wealth of data concerning both the genetic determinants of plant respiration and as we will see in the following a greater understanding of how this impacts metabolite levels and other aspects of plant physiology.

**Table 1:** List of natural variation studies assessing plant respiration

Species	Population size	Population type	Number of associations/QTL	Trait scored	Reference
Tomato (*S. lycopersicum × Solanum pennellii*)	6	Bi-parental (introgression lines)	N.A.	Transcript changes in respiratory enzymes in high Brix lines	(Baxter et al., 2005)
Barley (*Hordeum spontaneum*)	233	Bi-parental (F2 crossing population)	N.A.	Respiration—Gas exchange	(Poorter et al., 2005)
Melon (*Cucumis melo*)	27	Bi-parental (near isogenic lines)	None	Respiration rate	(Moreno et al., 2008)
Tomato (*S. lycopersicum × S. pennellii*)	76	Bi-parental (introgression lines)	27	Enzyme activity of the central carbon metabolism	(Steinhauser et al., 2011)
Melon (*C. melo)*	1131	Bi-parental (recombinant inbred lines)	1	Respiration—fruit ripening	(Ríos et al., 2017)
Tomato (*S. lycopersicum × Solanum pennellii)*	71	Bi-parental (introgression lines)	33	Dark respiration	(de Oliveira Silva et al., 2018)
Rice (*Oryza sativa)*	206	GWAS	1	Dark respiration	(Qu et al., 2020)
Winter wheat (*Triticum aestivum*)	276	GWAS	Multiple	Root respiration (CO_2_ efflux)	(Guo et al., 2021)
Rice (*O. sativa*)	432	MAGIC	Multiple	Grain yield—dark respiration	(Xu et al., 2021)
Melon (*C. melo*)	N.A.	Bi-parental (introgression lines)	N.A.	Respiration rate	(Zarid et al., 2021)

## Natural variation in the levels of respiratory metabolites

Whilst the number of studies directly assessing the rate of respiration is relatively rare those, which determine the levels of respiratory metabolites, are legion. This is probably largely due to the establishment of metabolomics techniques, which render the determination of metabolite levels to be accurately assessed across huge populations. We were able to list over 40 papers in which the levels of largely TCA cycle intermediates were determined (Supplemental Table 1). This list is not comprehensive, but we have tried to make it representative of the species analyzed. The approach of metabolomics has been extensively defined elsewhere (Fiehn, 2002; Saito and Matsuda, 2010; Alseekh and Fernie, 2018), so we will not detail it here suffice to say that the approach has been adapted to be used at scale and can thereby be used to provide accurate screening of metabolite levels across large populations providing certain precautions are taken (Alseekh et al., 2021a). Given how many studies have evaluated respiratory metabolites, we will not discuss them all individually but rather use a few case studies to highlight the power of this approach.

The levels of respiratory metabolites, mainly organic acids, and their composition is crucial for fruit acidity and thereby improving anthropocentric desired fruit quality. The initiation of the detection of respiratory metabolite QTL (rmQTL) was partially serendipitous due to their good coverage in GC-MS-based metabolomics. For this reason, a plethora of information is available across crops including tomato (Causse et al., 2004; Tieman et al., 2006, 2017; Liu et al., 2016b; Bauchet et al., 2017; Zhao et al., 2019; Çolak et al., 2020), sweet melon (Cohen et al., 2012; Freilich et al., 2015), eggplant (Toppino et al., 2016), strawberry (Vallarino et al., 2019; Barbey et al., 2021), blackcurrants (Abreu et al., 2020), apple (Liao et al., 2021), apricot (Dondini et al., 2022), and grapevine (Flutre et al., 2022). Due to space constraints, only exemplary discovery of rmQTL in the case of tomato will be presented, since it is one of the best-studied crop species. QTL mapping in tomato was largely centered on the introgression of *Solanum pennellii* (LA0716) to the background of *S. lycopersicum* (cv. M82) by Eshed and Zamir (1994). This particular IL population was first screened for fruit compositional analysis using enzymatic assays by Causse et al. (2004), wherein the authors identified 63 genes involved in the TCA cycle regulation, including citrate synthase, aconitase, isocitrate dehydrogenase, succinate dehydrogenase, as well as malate dehydrogenase. Inhibition of the latter is being demonstrated to result in enhanced CO_2_ assimilation rates and photosynthetic activity (Nunes-Nesi et al., 2005). Out of the eight rmQTL detected for citric acid previously, Tieman et al. (2006) could validate four, while reporting a novel rmQTL on chromosome 8. Concurrently, Schauer et al. (2006) demonstrated a strong correlation of respiratory metabolites to morphological traits in addition to a rmQTL for malate harboring a vacuolar pyrophosphatase. To determine the mode of inheritance, a follow on lines heterozygous for the introgression were analyzed by Schauer et al. (2008) indicating mostly dominant and additive inheritance for the majority of the rmQTL (>50%) with low to intermediate hereditability. Particularly interesting for fruit flavor, IL 4–4 was identified to display multiple changes in the levels of respiratory metabolites and was further narrowed by using sub-ILs providing higher resolution for the introgression and comprehensive profiling of metabolites and transcripts to give insight into fruit biochemistry (Liu et al., 2016b). After several field experiments on the ILs, Alseekh et al. (2017) explored the genetic plasticity in terms of metabolite canalization using the coefficient of variance over the different environments to determine canalization metabolite QTL (cmQTL). The detected cmQTL, including those for malate, citramalate, and 2-oxoglutarate on chromosome 10, were further cross-validated using a BIL population and most overlap with known rmQTL. Next to *S. pennellii*, other donor species like *S. pimpinellifolium* were used by Çolak et al. (2020) to identify rmQTL in addition to the previously reported ones. Contemporaneous to using the bi-parental populations, several GWAS were performed using modern, heirloom, and wild accessions of tomato to determine QTL associated with fruit aroma. To this end, the evaluation of consumer panels integrated into the chemical composition revealed flavor QTL, as well as rmQTL for malic acid and citric acid (Tieman et al., 2017). Furthermore, two associations were reported for fruit acidity, one being a malate transporter coding gene (Bauchet et al., 2017).

Besides fruit quality, other physiology-related components such as plant growth and development, are strongly associated with the levels of respiratory metabolites and have also been dissected for QTL discovery. In this context, major emphasis is given to grain crops such as maize, for which the metabolic levels of an RIL population were analyzed at different developmental stages revealing mQTL harboring genes for 2-oxoglutarate dehydrogenase, malate dehydrogenase, aconitase, succinate dehydrogenase, phosphoenolpyruvate carboxylase, and thioredoxin H-type across tissues and a pairwise epistatic interaction for >25.9% (Wen et al., 2015). In addition, an IL population derived from a cross between cultivated maize and its wild relative teosinte as donor was subjected to mQTL analysis and correlation analysis revealed among others positive correlation of yield to malate and 2-oxglutarate (Li et al., 2019). Other populations such as NAM population were likewise investigated for central carbon metabolism with resolution possible to the single gene level. With this approach, authors were able to identify carbonic anhydrase and a malate transporter as important components of yield (Zhang et al., 2015).

Besides crop species, traditionally, the model organism *A. thaliana* was investigated in a plethora of studies profiling the levels of respiratory metabolites in various bi-parental populations (Keurentjes et al., 2008; Lisec et al., 2008; Rowe et al., 2008; Fusari et al., 2017; Knoch et al., 2017) and GWA panels (Chan et al., 2010; Wu et al., 2016; Zhu et al., 2022). Additionally to conventional approaches to identify rmQTL, other statistical approaches were integrated, such as the study of Rowe et al. (2008), in which the authors identified that the majority of the central carbon variation was controlled by epistatic interactions. Further comparison with wild accessions demonstrated selective sweeps of previously showing genotype-metabolite associations indicating that evolutionary constraints are limiting metabolic variation (Chan et al., 2010).

More recently, emphasis has been given to studies indicating the outperformance of genomic prediction by metabolite based prediction model for example rice yield under drought stress (Melandri et al., 2022) and enhancing fruit flavor (Colantonio et al., 2022) highlighting the importance of respiratory metabolite levels and composition for multiple aspects of plant (stress) physiology.

## Respiratory metabolite fingerprints caused by natural trait variation

A yet more indirect assessment of respiration is provided by the analysis of traits that are affected by respiratory metabolites. As can be seen in Table 2, the vast majority of these are concerned with root exudation as such they are consistent with the findings that saturation transgenic experiments targeting the TCA cycle in tomato (van der Merwe et al., 2009, 2010). These studies have, however, been carried out in a broad range of species encompassing Arabidopsis, tomato, rice, maize, lentil (*Lens culinaris*), and common bean (*Phaseolus vulgaris*). Perhaps unsurprisingly, the first of these studies was focused on Arabidopsis—specifically on malate efflux in supporting aluminum (Al) tolerance. In these studies, Hoekenga and coworkers used RILs resulting from a cross between Landsberg erecta and Col-0 and examined malate exudation mediated Al tolerance. It is known that Al-activates the release of Al-binding organic acids from the root tip (Meyer et al., 2010), preventing uptake into the primary site of toxicity. In their analysis two major loci, which explain approximately 40% of the variance in Al tolerance observed among RILs derived from Landsberg erecta (which is Al-sensitive) and Columbia (which is Al-tolerant). In addition, on characterizing the mechanism by which tolerance is achieved, they found that the two QTL cosegregate with an Al-activated release of malate from Arabidopsis roots with one of these loci subsequently being found to contain the Al-activated malate transporter 1 (Hoekenga et al., 2003, 2006). This gene was additionally found in an independent GWAS study on Al and proton stress (Nakano et al., 2020a, 2020b). The homolog of this gene was subsequently characterized in a GWAS study to be responsible for fruit malate content in tomato (Tieman et al., 2017; Ye et al., 2017). Other studies on Al tolerance in lentil and common bean resulted in the identification of a major QTL for malate secretion and candidate gene including the Al-activated malate transporter and a multidrug and toxic compound extrusion gene in lentil and common bean, respectively (Ambachew and Blair, 2021; Singh et al., 2021).

**Table 2:** List of natural variation studies assessing traits influenced by respiratory metabolites

Species	Population size	Population type	Number of associations/QTL	Trait scored	Reference
Thale cress (*Arabidopsis thaliana*) (Ler × Col-0)	100	Bi-parental (recombinant inbred lines)	1	Aluminum tolerance (Malate efflux)	(Hoekenga et al., 2003, 2006)
Tomato (*S. lycopersicum*)	272	GWAS	1	Fruit malate content and Aluminum tolerance—organic acids	(Ye et al., 2017)
Maize (*Z. mays*)	338	Bi-parental (recombinant inbred lines)	multiple	Phosphorus deficiency—organic acids	(Luo et al., 2019)
Thale cress (*Arabidopsis thaliana*)	200	GWAS	300	Aluminum and Proton tolerance—organic acid exudation	(Nakano et al., 2020a, 2020b)
Lentil (*Lens culinaris*)	146	Bi-parental (recombinant inbred lines)	1	Aluminum tolerance—organic acid secretion	(Singh et al., 2021)
Common Bean *(Phaseolus vulgaris*)	227	GWAS	multiple	Al toxicity—organic acids	(Ambachew and Blair, 2021)
Thale cress (*Arabidopsis thaliana*)	387	GWAS	22	Flooding tolerance	(Meng et al., 2022)

More recently, RILs of maize were screened for the ability to grow in phosphorus deficient soils since organic acid secretion represents an important strategy to sequester phosphate (Nunes-Nesi et al., 2013). In this study, metabolite profiling was utilized alongside phenotyping to identify metabolites, which correlated with high phosphate use efficiency perhaps surprisingly the levels of alanine and isoleucine correlated best with this trait (Luo et al., 2019). That said, both of these metabolites are well documented to be part of the response to energy stress (Obata and Fernie, 2012; Zhu et al., 2022) so perhaps it is not so surprising that they, rather than the organic acids themselves, correlate better—particularly given the fact that the organic acids are exuded from the roots. Besides Al toxicity, other abiotic cues, e.g. flooding, lead to a signal transduction cascade altering metabolic fluxes, as it was shown in a current study by Meng et al. (2022), where authors identified *aconitase 3* (*ACO3*) and demonstrated co-localization of *ACO3* with the master regulation of retrograde signaling *Arabidopsis NAC domain-containing protein 17* leading to submergence induced metabolic priming in *ACO3* overexpression lines.

## Conclusions and future perspectives

To summarize, using natural variance as a means of dissecting the genetic architecture of respiration is beginning to complement transgenic and gene-editing-based work. That said, as we have illustrated, most of the studies to date look into traits associated with respiration rather than respiration per se. Considering both approaches, breeding populations which maximizes allelic diversity in a population allowing high resolution and GWAS having the potential to resolve many limitations such as population structure, gene–environment interactions, and missing heredity by gold standard genome sequences, will likely ultimately complementarily aid in defining the genetic determinants which influence respiratory related traits. Further, given the vast number of breeding populations as well as panels of accessions that can be used in GWASs alongside improvements of approaches to measure respiration in high-throughput with the recently established miniaturized methods described above which are adaptable to high-throughput studies, it is unlikely that this will remain the case for much longer. We anticipate that we will shortly be able to access the vast amount of data required to reveal the underlying genetic determinants of plant respiration. A further advantage that can be envisaged is the fact that such data could be integrated with the many other phenotypes that have been recorded for these populations, which will likely greatly enhance our knowledge of how the natural variation of respiration impacts other aspects of plant function (see Outstanding Questions).

## Advances

- Recent innovations in high-throughput methods to determine respiration rates have reignited screening highly diverse natural populations in the field of ecophysiology.
- Over the last decades, a vast excess of rmQTL for a plethora of crops has been identified due to improvements in metabolomics techniques.
- Metabolic prediction models have emerged that outperform genomic predictions on crop performance with several respiratory metabolites being major predictors.

## Outstanding questions

- Does natural diversity in respiration affect plant performance?
- If so, to what degree does it interconnect with other physiological aspects relevant to crop physiology?
- How did diversity in respiration emerge from an evolutionary perspective?

## Supplemental data

The following materials are available in the online version of this article.

**Supplemental Table S1.** List of natural variation studies assessing levels of respiratory metabolites in plants.

## Supplementary Material

kiac593_Supplementary_Data
