# Supplementary material for: Natural variation of respiration-related traits in plants
Source: Plant Physiol. 2022 Dec 22;191(4):2120–32. doi: 10.1093/plphys/kiac593 (PMC10069898; doi:10.1093/plphys/kiac593)
Supplement: kiac593_Supplementary_Data [file kiac593_supplementary_data.pdf]

Supplemental Table I List of natural variation studies assessing plant respiration.

Species	Population size	Population type	Number of Associations/QTL	Trait scored	Reference
Strawberry (*Fragaria × ananassa*)	193	Bi-parental (F2 offspring)	7	Primary metabolites – organic acids	(Moing et al., 2004)
Tomato (*Solanum lycopersicum x Solanum pennellii*)	75	Bi-parental (introgression lines)	multiple	Fruit weight, Primary metabolites – organic acids	(Causse et al., 2004)
Tomato (*Solanum lycopersicum x Solanum pennellii*)	76	Bi-parental (introgression lines)	93	Yield, Primary metabolites – organic acids	(Schauer et al., 2006)
Tomato (*Solanum lycopersicum x Solanum pennellii*)	74	Bi-parental (introgression lines)	multiple	Flavor compounds – organic acids	(Tieman et al., 2006)
Thale cress (*Arabidopsis thaliana*) (Col-0 x C24)	429 + 97	Bi-parental (recombinant inbred lines + introgression lines)	157	Primary metabolites – organic acids	(Lisec et al., 2008)
Thale cress (*Arabidopsis thaliana*) (Bay x Sha)	210	Bi-parental (recombinant inbred lines)	multiple	Primary metabolites – organic acids	(Rowe et al., 2008)
Tomato (*Solanum lycopersicum x Solanum pennellii*)	N.A.	Bi-parental (introgression lines; heterozygous for the introgression)	multiple	Primary metabolites – organic acids	(Schauer et al., 2008)
Thale cress (*Arabidopsis thaliana*) (Ler x Cvi)	160	Bi-parental (recombinant inbred lines)	multiple	Carbon metabolism	(Keurentjes et al., 2008)
Thale cress (*Arabidopsis thaliana*)	96	GWAS	32	Primary metabolites – organic acids	(Chan et al., 2010)
Maize (*Zea mays*)	289	GWAS	multiple	Primary metabolites – organic acids	(Riedelsheimer et al., 2012)
Melon (*Cucumis melo*)	99	Bi-parental (recombinant inbred lines)	6	pH & Primary metabolites – organic acids	(Cohen et al., 2012)

Maize (*Zea mays*)	197	Bi-parental (recombinant inbred lines)	297	Primary metabolites – organic acids	(Wen et al., 2015)
Melon (*Cucumis melo*)	96	Bi-parental (recombinant inbred lines)	N.A.	Organic acids (flavor)	(Freilich et al., 2015)
Bread wheat (*Triticum aestivum*)	197	Bi-parental (double haploid lines)	2	Primary metabolites – organic acids	(Hill et al., 2015)
Maize (*Zea mays*)	5,000	Multi-parental (nested association mapping population 25 (200 RILs each crossing))	18	Primary metabolites – organic acids	(Zhang et al., 2015)
Eggplant (*Solanum melongena*)	156	Bi-parental (F2 backcrossed double haploid)	6	Primary metabolites – organic acids	(Toppino et al., 2016)
Tomato (*Solanum lycopersicum x Solanum pennellii*)	71	Bi-parental (introgression lines)	multiple	Primary metabolites – organic acids	(Rosental et al., 2016)
Rice (*Oryza sativa*)	280	Bi-parental (recombinant inbred lines)	multiple	Primary metabolites – organic acids	(Li et al., 2016)
Tomato (*Solanum lycopersicum x Solanum pennellii*)	75	Bi-parental (introgression lines)	1	Primary metabolites – organic acids (flavor)	(Liu et al., 2016)
Thale cress (*Arabidopsis thaliana*)	314	GWAS	36	Primary metabolites – organic acids	(Wu et al., 2016)
Tomato (*Solanum lycopersicum* and wild relatives)	300	GWAS	79	Primary metabolites – organic acids	(Bauchet et al., 2017)
Tomato (*Solanum lycopersicum x Solanum pennellii*)	68	Bi-parental (Backcrossed introgression lines)	multiple	Primary metabolites – organic acids	(Alseekh et al., 2017)
Thale cress (*Arabidopsis thaliana*) (Col-0 x C24)	393	Bi-parental (recombinant inbred lines)	157	Primary metabolites – organic acids	(Knoch et al., 2017)

Rice (*Oryza sativa*)	380	Bi-parental (recombinant inbred lines)	1	Primary metabolites – organic acids	(Daygon et al., 2017)
Perennial ryegrass (*Lolium perenne*)	325	F2 Bi-parental from 2 inbred lines	11	Primary metabolites – organic acids	(Foito et al., 2017)
Thale cress (*Arabidopsis thaliana*)	349	GWAS	2	Primary metabolites – organic acids	(Fusari et al., 2017)
Tomato (*Solanum lycopersicum* and wild relatives)	398	GWAS	multiple	Flavor – organic acids	(Tieman et al., 2017)
Maize (*Zea mays*)	513	GWAS	153	Primary metabolites – organic acids	(Wen et al., 2018)
Strawberry (*Fragaria × ananassa*)	95	Bi-parental (F1 progeny lines)	155	Primary metabolites – organic acids	(Vallarino et al., 2019)
Maize (*Zea mays x Zea Mexicana*)	191	Bi-parental (introgression lines)	multiple	Primary metabolites – organic acids	(Li et al., 2019)
Tomato (*Solanum lycopersicum x Solanum pennellii*)	76	Bi-parental (introgression lines)	118	Primary metabolites – organic acids	(Nunes-Nesi et al., 2019)
Tomato (*Solanum lycopersicum*)	775	GWAS	305	Primary metabolites – organic acids (flavor)	(Zhao et al., 2019)
Blackcurrant (*Ribes nigrum L.*)	125	Bi-parental (F1 segregation population)	multiple	Primary metabolites – organic acids	(Abreu et al., 2020)
Tomato (*Solanum lycopersicum x Solanum pimpinellifolium*)	N.A.	Bi-parental (backcrossed inbred lines)	14	Primary metabolites – organic acids	(Çolak et al., 2020)
Lettuce (*Lactuca sativa*)	189	GWAS	51	Primary metabolites – organic acids	(Zhang et al., 2020)
Strawberry (*Fragaria ananassa*)	213	GWAS	5	Primary metabolites – organic acids & volatile	(Barbey et al., 2021)

				organic compounds	
Apple (*Malus species*)	497	GWAS	multiple	Primary metabolites – organic acids	(Liao et al., 2021)
Tomato (*Solanum lycopersicum*)	163	GWAS	45	Fruit weight & primary metabolites – organic acids	(Zhao et al., 2022)
Apricot (*Prunus areniaca*)	118	Bi-parental (F1 cross)	multiple	Primary metabolites – organic acids	(Dondini et al., 2022)
Grape vine (*Vitis vinifera*)	279	GWAS	multiple	Primary metabolites – organic acids	(Flutre et al., 2022)
Thale cress (*Arabidopsis thaliana*)	252	GWAS	215	Primary metabolites – organic acids under darkness	(Zhu et al., 2022)
